# Differential homeostatic reorganization during high-altitude trekking: multisystemic adaptations and organ-specific resilience

**DOI:** 10.1007/s00421-026-06410-7

**Published:** 2026-08-29

**Authors:** Manuel Marzola, Matteo De Angelis, Lucrezia Lacordara, Ariadna Micaela Lucero, Gerardo Bosco, Simona Mrakic-Sposta, Vittore Verratti

**Affiliations:** 1 Department of Medicine and Aging Sciences, University “G. d’Annunzio” of Chieti-Pescara, Chieti, Italy; 2 School of Medicine and Health Sciences, University “G. d’Annunzio” of Chieti-Pescara, Chieti, Italy; 3 Department of Science, University “G. d’Annunzio” of Chieti-Pescara, Chieti, Italy; 4 Chronophysiology Lab, Institute for Biomedical Research (BIOMED), Catholic University of Argentina (UCA), and National Scientific and Technical Research Council (CONICET), Buenos Aires, Argentina; 5 Institute of Clinical Physiology, National Research Council (IFC-CNR), Milan, Italy; 6 Italian Society of Mountain Medicine, Padua, Italy

**Keywords:** High-altitude, Cardiovascular adaptation, Renal function, Lipid metabolism, Energy deficit, Organ Resilience

## Abstract

**Introduction:**

High-altitude exposure combined with physical activity triggers complex systemic adaptations. However, multidisciplinary longitudinal investigations into these multiorgan responses remain limited. This prospective observational study aimed to investigate the cardiovascular, metabolic, renal, and hepatic responses to a subacute high-altitude expedition.

**Methods:**

21 Caucasian participants (14 men and 7 women) participated during a Himalayan trek expedition up to 5050 m. Measurements included: cardiovascular parameters (HR, BP) evaluated at five points (baseline (T0), intermediate ascent (T0a), peak (T1), intermediate descent (T1a), post-trek (T2)); comprehensive biochemical parameters (renal, hepatic, and lipid markers) collected at four time points: baseline (T0), peak altitude (T1), post-trek (T2), and a several-month follow-up (T3); body weight at three points (T0, T1, T2) and dietary intake.

**Results:**

Ascent induced significant increases in HR and BP, with DAP remaining elevated during descent. At peak altitude, the A/G ratio decreased while U/C and GFR increased, alongside reductions in creatinine and body weight. Despite a severe energy deficit (44–52% below predicted TDEE) and high lipid intake, total cholesterol, LDL, and HDL decreased significantly. Notably, markers of hepatic and muscular injury (AST, ALT, ALP, CK) remained stable throughout the expedition.

**Conclusion:**

Subacute high-altitude trekking imposes a selective physiological strain rather than uniform systemic stress. The body prioritizes vital hemodynamic and renal adaptations; at the same time, despite a negative energy balance, it exhibits organ-specific resilience, as demonstrated by hepatic and muscular stability. Finally, the complete recovery of markers at follow-up confirms these multisystemic shifts are highly adaptive and non-pathological.

## Introduction

High-altitude exposure triggers systemic adaptations to counteract the reduced oxygen availability in the tissues (West et al. 2012). Acute responses include hyperventilation, increased heart rate and elevated blood pressure induced by peripheral chemoreceptor activation (Smith et al. 1986) and an increase in the sympathetic system (Hainsworth et al. 2007). While heart rate tends to reduce after approximately 10 days of exposure, the increase in blood pressure persists (Mallet et al. 2023).

This sympathetic activation affects not only cardiac function but also renal and vascular activity (Hainsworth et al. 2007; Wang et al. 2022). In combination with respiratory alkalosis induced by hypocapnia, it contributes to the acute modulation of the renin–angiotensin–aldosterone System (RAAS), followed within hours by renal bicarbonate excretion, diuresis and natriuresis (San et al. 2013; Wang et al. 2022). These responses lead to hemoconcentration and thereby an increase in hematocrit and blood viscosity (Goldfarb-Rumyantzev and Alper 2014). As a result, the renal system plays a crucial role in maintaining hydro-electrolytic homeostasis and in the chronic adaptation to hypoxia.

Reorganization of metabolic (Tam et al. 2016), hormonal (Verratti et al. 2017), musculoskeletal (Bosco et al. 2010) and immune systems (Morabito et al. 2016) also occurs in response to high-altitude hypoxia.

The reduction in oxidative capacity combined with an increase in oxidative stress leads to an enhanced glycolytic capacity, elevated triglyceride (TG) levels, reduced lipoprotein lipase activity, and suppression of de novo lipogenesis, allowing for the maintenance of adequate energy production. These metabolic changes are negotiated by transcriptional mechanisms through HIF-1/2α (Rankin et al. 2009) and influenced by catecholamine activity (Young et al. 1989). Nevertheless, the increased energy cost of exercise and the hypoxia-induced elevation in metabolic rate result in a negative energy balance, leading to a reduction in body weight, fat mass, and, especially with prolonged exposure, skeletal muscle mass loss (Dünnwald et al. 2019; Sitko et al. 2019).

High-altitude exercise further exacerbates oxidative stress by increasing reactive oxygen species (ROS) production, particularly during intense exercise above 3000 m (Miller et al. 2013; Quindry et al. 2016). Skeletal muscle represents a principal source of ROS production, making it susceptible to oxidative stress injury (Simon-Ugron et al. 2012), as reflected by higher creatine kinase (CK) levels after hypoxic exercise (Wilber et al. 2000). In addition to skeletal muscle involvement, high-altitude hypoxic exercise may exacerbate stress in additional organs (Mrakic-Sposta et al. 2022), increasing the risk of cellular injury. In the liver, this may be manifested by elevations in aspartate aminotransferase (AST) and alanine aminotransferase (ALT), reflecting hepatocellular impairment (Barnholt et al. 2006; Soliman et al. 2022).

While physiological responses to high altitude are well-documented, most research has focused primarily on the oxygen transport system and other physiological systems remain underexplored. In particular, there is a lack of multidisciplinary studies investigating how the body adapts to intermediate altitudes during recreational exercise (Reid et al. 2025).

In this context, a prospective observational study was conducted to investigate the physiological and biochemical responses to intermediate high-altitude exposure combined with physical activity. The study aimed to analyze cardiovascular parameters, metabolic regulation, organ-specific biomarkers, dietary intake, and body weight changes to provide a comprehensive evaluation of systemic adaptations to high-altitude physical activity.

## Methods

### Participants

**Fig. 1:**
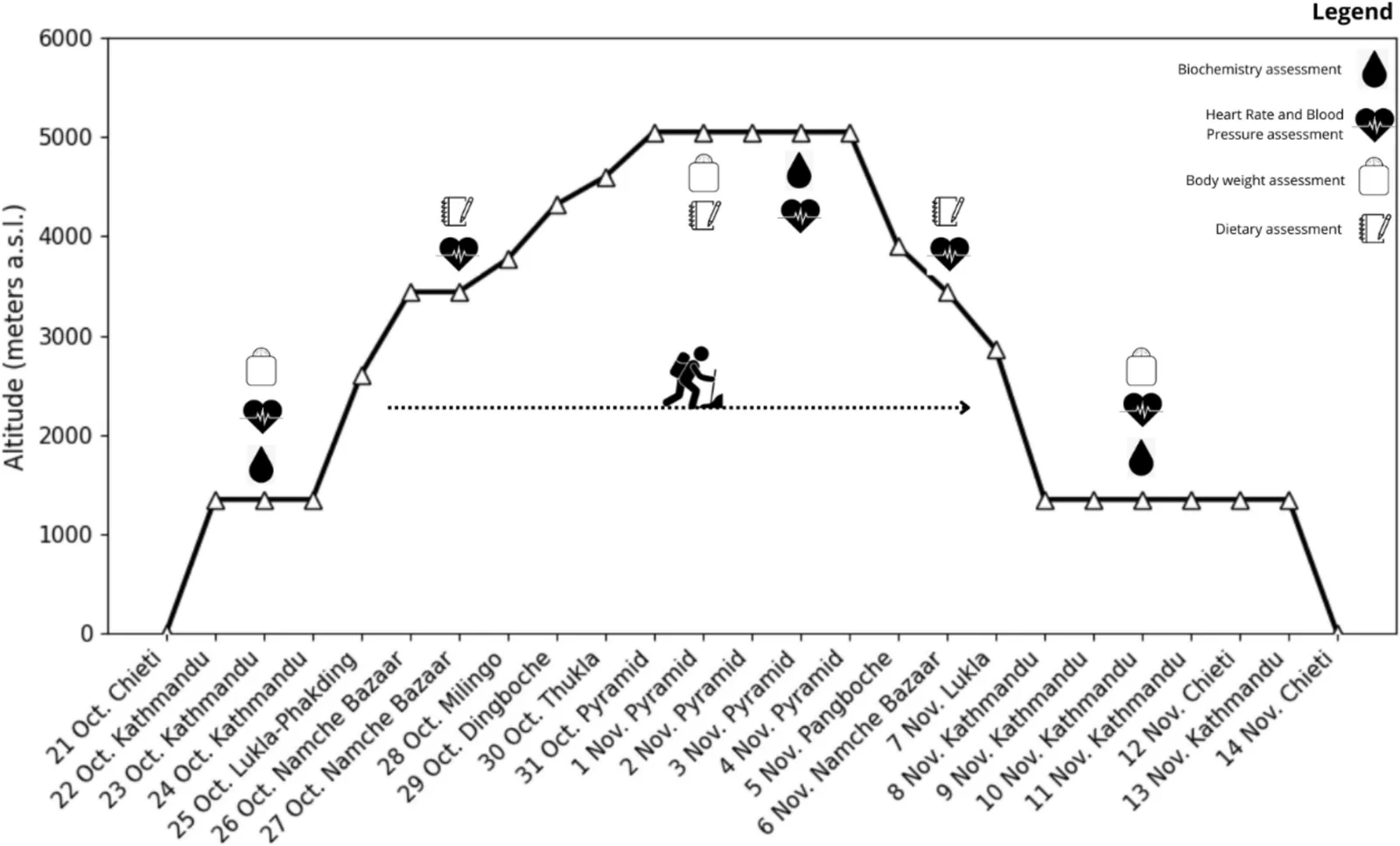
Timeline of data collection points during the Himalayan expedition (5050 m) showing altitude progression and sampling schedule

A total of 21 Caucasian participants (14 men and 7 women; 36.9 ± 12.8 years, range 21–56; BMI 24.1 ± 3.11 kg/m^2^, range 18.9–31.3) participated in a scientific expedition “Pyramid 24 exploration and physiology, multisystem and multiorgan responses to hypoxia in human adaptation of different geo-origin” in Nepal, from 23 October to 14 November 2024. During this period, the mountaineers were engaged in regular trekking activities to reach the Pyramid International Laboratory (5050 m) (Fig. 1 shows the trek time lines of the expedition and different time points). The expedition involved a trekking route of 121 km with a total altitude variation of 6,521 m. On average, the trekkers spent 6–8 h walking daily.

Before the expedition, a qualified medical professional conducted a medical screening. The presence of diabetes mellitus, significant cardiac or respiratory disease, or hypertension constituted exclusion from the study.

The expedition was supervised by a medical doctor specializing in altitude medicine. To ensure a standardized pharmacological baseline and facilitate acclimatization among all participants, a prophylaxis protocol of 125 mg acetazolamide twice daily was implemented throughout the ascent. NSAIDs were used exclusively for symptomatic treatment of headaches.

Written informed consent was obtained from all individuals. This research was approved by the Ethics Review Board of the Nepal Health Research Council (NHRC, ref. no. 1084). All study procedures conformed to the ethical standards set by the Declaration of Helsinki and its subsequent amendments.

### Biochemistry assessment

Blood samples were collected four times: at the baseline altitude in Kathmandu (T0), at the peak altitude (Pyramid Laboratory) (T1), on return to Kathmandu (T2), and during follow-up in Italy (Chieti) (T3) after several months. The temporal framework was delineated for the purpose of conducting a comprehensive analysis of the acute, sub-acute responses during hypobaric hypoxia and the immediate and long-term reversibility after high altitude exposure.

Serum albumin (Alb), total protein (TP), globulins (Glob), albumin-to-globulin ratio (A/G ratio), aspartate aminotransferase (AST), alanine aminotransferase (ALT), alkaline phosphatase (ALP), amylase (AMY), creatine kinase (CK), creatinine (Crea), estimated glomerular filtration rate (GFR), urea, urea-to-creatinine ratio (U/C), glucose (GLU), total cholesterol (TC), triglycerides (TG), high-density lipoprotein (HDL), and low-density lipoprotein (LDL) were measured.

Blood markers were analyzed at the point of care using a microfluidics-based Emoanalyzer (VChemy S®, Voden, Italy). Blood samples (100 μL) were collected via capillary sampling from the fingertips and introduced into the reacting rotors VChemy LIPID PANEL S® to be analyzed through Emoanalyzer. Emoanalyzer was automatically calibrated at each start and in case of error in the sampling and/or collection, analysis was repeated.

Capillary blood sampling combined with Point of Care (PoC) analysis represents an effective alternative to standard laboratory methods, which involve complex procedures such as sample centrifugation and strict cold-chain preservation. Those procedures in high-altitude settings are logistically unfeasible and may compromise validity. To improve feasibility while maintaining validity and reproducibility, PoC offers a suitable alternative, as supported by the literature (Cao et al. 2017; Fontaine and Saez 2023), which demonstrated concordance with standard venous laboratory analyses (Cao et al. 2017; Fontaine and Saez 2023) regarding validity and reproducibility.

The specific biochemical panel was selected to investigate physiological responses influenced by stress induced by hypoxia, physical exercise, and energy deficit. The hemodynamic and metabolic systems are highly metabolically active and play a fundamental role in maintaining homeostasis under stress, representing a potentially vulnerable target (Evans et al. 2020; Mallet et al. 2023). Specifically, renal markers (urea, creatinine, eGFR, and U/C ratio) were included to investigate renal hemodynamic responses. Hepatic and muscular enzymes (AST, ALT, ALP, and CK), alongside protein-related markers (albumin, total proteins, globulins, and the A/G ratio), were included to evaluate organ-specific stress, tissue damage and metabolic reorganization. Metabolic and lipid parameters (glucose, triglycerides, total cholesterol, LDL, and HDL) were selected to characterize alterations in energy metabolism during high-altitude trekking.

Capillary blood samples were collected in the morning; however, because we could not guarantee a strict fasting status for all participants, glucose (GLU), triglycerides (TG), and amylase (AMY) were excluded from the analysis to mitigate potential bias related to differences in sample collection timing.

### Heart rate and blood pressure assessment

Heart rate and blood pressure were measured daily during the ascent and descent phases of the study by an oscillometric electronic device (Omron M6 [HEM-7001-E], Omron Healthcare). Measurements were taken before dinner and in controlled conditions after a 10-min seated rest. Three measurements were taken, and the median value was recorded. To examine the acute and subacute phases of adaptation to hypobaric hypoxia during ascent and descent, a total of five points were considered: before (T0), at peak altitude (T1), after (T2), and two intermediate points during ascent (T0a) and descent (T1a) at Namche. These time points were strategically selected during days of rest or low physical exertion to minimize confounding variables and ensure a more accurate representation of cardiovascular sensitivity to high-altitude exposure.

### Body weight assessment

The body weight of the participants was measured on three occasions (T0-T2) in order to obtain baseline, intermediate, and final trek measurements. The second day was selected for logistical reasons. The measurement was taken in the morning in a fasting state; the participants were wearing light clothing and no shoes, and an automatic balance (Seca, Italy) was used.

### Dietary and total daily energy expenditure assessment

Dietary plans were not defined before the expedition. After receiving specific instructions, the participants documented everything they ate or drank, including supplements, using a food diary organized into seven daily intake moments (three main meals and four snacks). These records were compiled on non-consecutive days during the trek with an interval of several days. Food diaries were compiled on three non-consecutive days during the trek: at Namche during ascent (T0a), at the peak altitude reached (T2), and again in Namche during the descent (T1a). The temporal dimension was selected as a means of quantifying the various phases of trekking: ascent, peak altitude, sojourn and descent. For each item consumed, food, drink, prepared dish, or supplement, the participants reported the exact time and location of intake, along with a detailed description and the amount consumed. Portion sizes were estimated using a dedicated photographic atlas. All completed diaries were reviewed by a trained field researcher, who subsequently transferred the information into a customized online platform, the Food Consumption Database (FOODCONS). This tool was used for data coding and processing, converting each recorded food item into grams and into its corresponding energy intake (EI) and lipid-related variables (total fat, cholesterol, SFA, and PUFA). The software provides a full nutrient profile for each food item; however, in the present study, only total energy intake, total fat, cholesterol, saturated fatty acids (SFA), and polyunsaturated fatty acids (PUFA) were extracted and included in the analyses. These dietary variables were used to evaluate associations with biochemical and metabolic parameters and with high-altitude exposure. The software FOODCONS and all its associated resources, including food composition databases, paper-based diary templates, and the photographic atlas, were developed by the *Research Center for Food and Nutrition of the Council for Agricultural Research and Economics* (CREA—Food and Nutrition) (https://www.crea.gov.it/en/web/alimenti-e-nutrizione) for use in Italian food consumption surveys and were specifically adapted for this study.

Total Daily Energy Expenditure predicted (TDEEp) was estimated using a MET-based time–activity model, assigning 7.0 METs to daily hiking with a backpack (5 h·day⁻^1^) and 1.0 MET to the remaining resting time of the day (Herrmann et al. 2024).

### Statistical analysis

Statistical analyses were performed using Jamovi (v. 2.3.28).

Linear Mixed Models (LMM) were used to analyze changes in biochemical, cardiovascular, and anthropometric parameters across time points. Time point was entered as a fixed effect, and the parameter under investigation was set as the dependent variable. To account for the repeated-measures design and inter-individual variability, participant ID was included as a cluster variable (random intercept). Sex was not included as a factor in the models.

Model assumptions were rigorously verified through the Shapiro–Wilk test and visual inspection of Q-Q plots for normality assessment, and through residual plots analysis for homoscedasticity and linearity checks.

To account for multiple comparisons, parameters were grouped into biochemical (m=15) and cardiovascular (m=4) domains, with p-values adjusted via the Holm-Bonferroni procedure. Post hoc tests were performed only for significant main effects (adj-p < 0.05) using Bonferroni correction. Effect sizes were estimated using marginal R-squared (R^2^m).

A descriptive and qualitative analysis was conducted for nutritional data. Energy intake (EI) and macronutrient distribution (Lipids, SFA, PUFA, Cholesterol) were expressed as Mean ± SD and compared against estimated Total Daily Energy Expenditure (TDEE) and international nutritional limits.

All graphical representations were generated using GraphPad Prism (v. 8.4.3).

## Results

Significant multisystemic variations emerged in response to hypobaric hypoxia associated with physical activity.

Heart rate (HR) and mean arterial pressure (MAP) showed significant changes across different time points (HR: p < 0.004; R^2^m = 0.316; MAP: p < 0.004; R^2^m = 0.166), as did systolic arterial pressure (SAP) and diastolic arterial pressure (DAP) (SAP: p = 0.004; R^2^m = 0.0578; DAP: p < 0.004; R^2^m = 0.247), as reported in Table 1.

**Table 1:** Marginal R^2^, p-values, and Holm-Bonferroni adjusted p-values for cardiovascular responses (m=4)

	p-values	Adjusted p-values	R^2^m
HR	<0.001	<0.004	0.316
DAP	<0.001	<0.004	0.247
MAP	<0.001	<0.004	0.166
SAP	<0.001	<0.004	0.0578

*HR* Heart rate, *DAP* Diastolic blood pressure, *MAP* Mean arterial pressure, *SAP* Systolic blood pressure

**Fig. 2:**
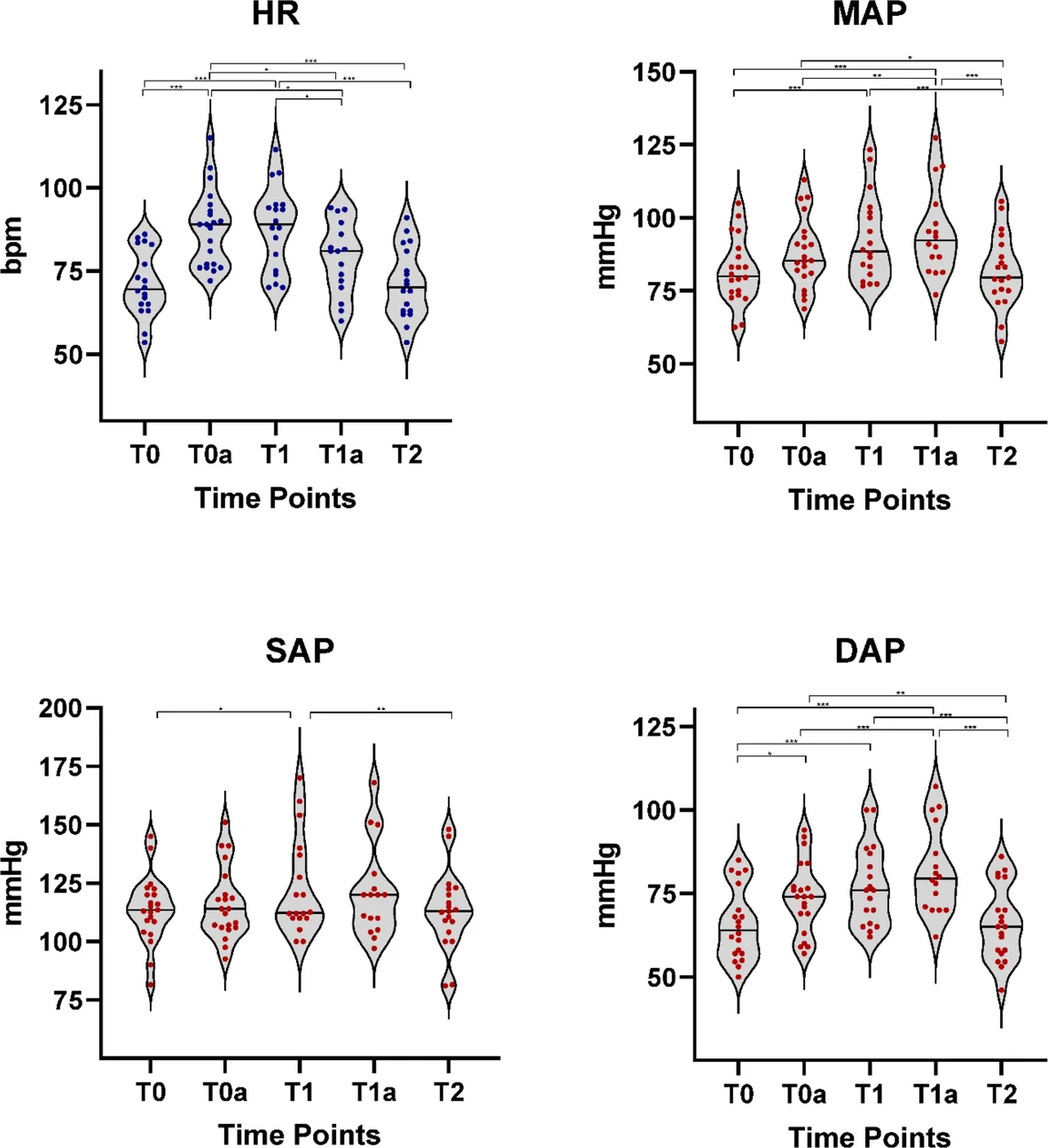
Cardiovascular responses across time points. *HR *heart rate (**a**), *MAP *mean arterial pressure (**b**), *DAP *diastolic blood pressure (**c**), *SAP *systolic blood pressure (**d**). ***: p<0.001; **: p<0.01; *: p<0.05

Post-hoc analysis revealed, for HR and DAP, a significant increase during ascent (T0 vs T0a: HR (bpm) = 72 ± 9.1 vs 87.7 ± 11.5, p < 0.001; DAP (mmHg) = 65.7 ± 10.8 vs 73.8 ± 10.8, p < 0.001; T0 vs T1: HR (bpm) = 72 ± 9.1 vs 87.9 ± 12.9, p < 0.001; DAP (mmHg) = 65.7 ± 10.8 vs 77.3 ± 11.9, p < 0.001). A significant decrease in HR was observed during descent (T1 vs T1a: 87.9 ± 12.5 vs 78.9 ± 10.9 bpm, p = 0.022; T1 vs T2: 87.9 ± 12.5 vs 71.4 ± 10.7 bpm, p < 0.001), whereas DAP remained elevated at the intermediate descent point and returned to baseline at T2 (T0 vs T1a: 65.7 ± 10.8 vs 81.9 ± 13.1 mmHg, p < 0.001; T1a vs T2:81.9 ± 13.1 vs 66 ± 11.5 mmHg, p < 0.001). SAP showed a significant increase at peak altitude compared with baseline (T0 vs T1: 113.1 ± 13.8 vs 123.2 ± 20.7 mmHg, p = 0.005), as well as during descent (T1a vs T2: 121.2 ± 19.8 vs 113.1 ± 16.9 mmHg, p = 0.043; T1 vs T2: 123 ± 20.7 vs 113.1 ± 16.9 mmHg, p = 0.004). Figure 2 shows the cardiovascular responses across time.

High-altitude exposure profoundly influenced the albumin–globulin ratio (A/G) resulting in a significant reduction at peak altitude (T0 vs T1: 2.00 ± 0.2 vs 1.6 ± 0.2, p < 0.001), which was maintained until T2 (T0 vs T2: 2.00 ± 0.2 vs 1.7 ± 0.2, p < 0.001) and returned to values similar to baseline at follow-up (T1 vs T3: 1.6 ± 0.2 vs 1.9 ± 0.2, p < 0.001).

Changes in the A/G ratio were driven by a significant reduction in albumin (Alb) levels (T0 vs T1: 4.7 ± 0.3 vs 4.4 ± 0.2 gr/dL, p < 0.001) and an increase in globulin (Glob) levels at T1 (T0 vs T1: 2.4 ± 0.3 vs 2.8 ± 0.3 gr/dL, p < 0.001). Both parameters decreased at T2 (Alb (gr/dL): T1 vs T2: 4.4 ± 0.2 vs 4.2 ± 0.2, p < 0.001; T0 vs T2: p < 0.001; Glob (gr/dL): T1 vs T2: 2.8 ± 0.3 vs 2.5 ± 0.3, p < 0.001). At follow-up, ALB significantly rose compared to T2 (T2 vs T3: 4.2 ± 0.2 vs 4.5 ± 0.2 gr/dL, p < 0.001) but was not able to return to the baseline value (T0 vs T3: 4.7 ± 0.3 vs 4.5 ± 0.2 gr/dL, p = 0.006). Instead, Glob returned to values comparable to baseline during follow-up (T1 vs T3: 2.8 ± 0.3 vs 2.4 ± 0.2 gr/dL, p < 0.001). Total protein (TP) levels were not markedly affected at high altitude but showed a significant decrease at T2 (T0 vs T2: 7.1 ± 0.5 vs 6.7 ± 0.7 gr/dL, p = 0.033; T1 vs T2: 7.2 ± 0.4 vs 6.7 ± 0.7 gr/dL, p = 0.002).

Urea (U) and the urea-to-creatinine ratio (U/C) similarly increased at peak altitude (U (mg/dL): T0 vs T1: 33.4 ± 7.0 vs 41.0 ± 10.1, p < 0.001; U/C: T0 vs T1: 36.0 ± 7.8 vs 55.0 ± 16.9, p < 0.001). The U/C ratio remained elevated at T2 (T0 vs T2: 36.0 ± 7.8 vs 48.0 ± 10.2, p = 0.002) and returned toward baseline at follow-up (T1 vs T3: 48.0 ± 10.2 vs 41.0 ± 9.4, p = 0.013), whereas U levels began to decrease at T2 and returned to baseline at follow-up (T1 vs T2: 41.0 ± 10.1 vs 36 ± 7.2 mg/dL, p= 0.043; T1 vs T3: 41.0 ± 10.1 vs 34.0 ± 6.9 mg/dL, p = 0.015). On the contrary, creatinine is reduced at high altitude (T0 vs T1: 1.0 ± 0.2 vs 0.8 ± 0.2 mg/dL, p = 0.004) and is maintained after trek (T0 vs T2: 1.0 ± 0.2 vs 0.8 ± 0.3 mg/dL, p = 0.003) and follow-up (T0 vs T3: 1.0 ± 0.2 vs 0.8 ± 0.2 mg/dL, p = 0.037). On the contrary, GFR increased at peak altitude (T0 vs T1: 99.1±18.4 vs 111 ± 17, p = 0.002) and higher values were maintained after the trek (T0 vs T2: 99.1± 18.4 vs 112.4 ± 16.3, p<0.001).

A significant reduction was observed in total cholesterol (TC), low-density lipoprotein (LDL), and high-density lipoprotein (HDL) during the trek (T0 vs T2: TC (mg/dL) = 192.7 ± 42.4 vs 155 ± 28.2, p < 0.001; LDL (mg/dL) = 112.4 ± 39.0 vs 74.0 ± 22.6, p < 0.001; HDL (mg/dL) = 50.9 ± 10.8 vs 43.0 ± 4.6, p = 0.001). The largest reduction occurred at peak altitude (T0 vs T1: TC (mg/dL) = 192.7 ± 42.4 vs 145 ± 27.2, p < 0.001; LDL (mg/dL) = 112.4 ± 39.0 vs 79.0 ± 22.0, p < 0.001; HDL (mg/dL) = 50.9 ± 10.8 vs 35.0 ± 6.4, p < 0.001).

Recovery patterns differed among lipid parameters. TC gradually started to increase and returned toward baseline from peak descent to follow-up (T1 vs T3: 145 ± 27.2 vs 187 ± 27.2 mg/dL, p < 0.001, T2 vs T3: 155 ± 28.2 vs 187 ± 27.2 mg/dL, p = 0.002), as did HDL (T1 vs T3: 35.0 ± 6.4 vs 51.0 ± 8.0 mg/dL, p < 0.001; T1 vs T2: 35.0 ± 6.4 vs 43.0 ± 4.6, p<0.001; T2 vs T3: 43.0 ± 4.6 vs 51.0 ± 8.0 mg/dL, p = 0.021). LDL remained reduced after trek and recovered significantly at T3 (T2 vs T3: 74.0 ± 22.6 vs 104.0 ± 17.4 mg/dL, p = 0.001; T1 vs T3: 79.0 ± 22.0 vs 104.0 ± 17.4 mg/dL, p = 0.02), although not to baseline levels (T0 vs T3: 112.4 ± 39.0 vs 104.0 ± 17.4 mg/dL, p = 0.172).

**Fig. 3:**
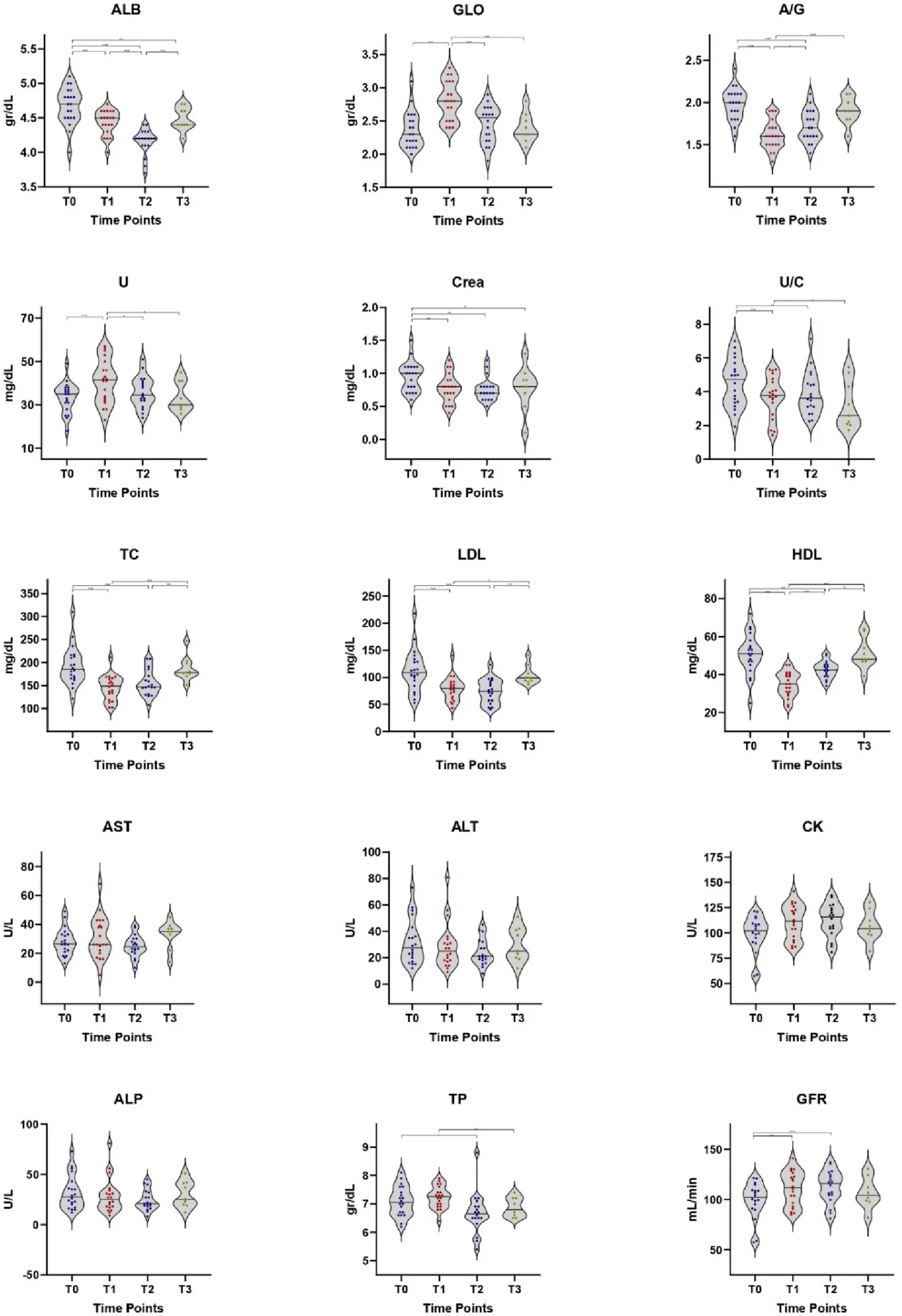
Biochemical parameters variations across time points. *ALB* albumin (**a**); *GLOB* globulin (**b**); *A/G* albumin/globulin ratio (**c**); *U* urea (**d**); *Cr* creatinine (**e**); *U/C* urea/creatinine ratio (**f**); *TC* total cholesterol (**g**); *LDL* low-density lipoprotein (**h**); *HDL* high-density lipoprotein (**i**); *AST* aspartate aminotransferase (**l**); *ALT* alanine aminotransferase (**m**); *CK* creatine kinase (**n**); *ALP* alkaline phosphatase (**o**); *TP* total proteins (**p**); *GFR* glomerular filtration rate (**q**). ***: p<0.001; **: p<0.01; *: p<0.05

AST (adj-p = 0.308, R^2^m = 0.061), ALT (adj-p = 0.228, R^2^m = 0.035), ALP (adj-p = 0.228, R^2^m = 0.034), and CK (p = 0.308, R^2^m = 0.035) did not show significant variations across the four time points. The original p-values, the adjusted p-values and marginal R-squared are reported in Table 2. Violin plots (Fig. 3) illustrate the longitudinal biochemical responses.

**Table 2:** Marginal R^2^, p-values, and Holm-Bonferroni adjusted p-values for biochemical parameters (m=15)

	p-values	Adjusted p-values	R^2^m
HDL	< 0.001	< 0.015	0.682
ALB	< 0.001	< 0.015	0.467
A/G	< 0.001	< 0.015	0.417
GLOB	< 0.001	< 0.015	0.316
U/C	< 0.001	< 0.015	0.290
TC	< 0.001	< 0.015	0.279
LDL	< 0.001	< 0.015	0.253
U	< 0.001	< 0.015	0.129
Cr	< 0.001	< 0.015	0.118
GFR	< 0.001	< 0.015	0.116
TP	0.002	0.015	0.153
ALP	0.057	0.228	0.034
ALT	0.073	0.228	0.035
CK	0.154	0.308	0.035
AST	0.163	0.308	0.061

*ALB* Albumin, *GLOB* Globulin, *A/G* Albumin/globulin ratio, *U* Urea, Cr Creatinine, *U/C* Urea/creatinine ratio, *TP* Total proteins, *TC* Total cholesterol, *LDL* Low-density lipoprotein, *HDL* High-density lipoprotein, *CK* Creatine kinase, *AST* Aspartate aminotransferase, *ALT* Alanine aminotransferase, *GFR* Estimated glomerular filtration rate, *ALP* Alkaline phosphatase

The high-altitude trek had a significant effect on participants’ body mass (p < 0.001, R^2^m = 0.002). A pronounced reduction occurred during the ascent (T0 = 73.8 ± 3.11 vs. T1 = 72.2 ± 3.11 kg, p < 0.001), and body mass remained below baseline for several days after the trek ended (T1 = 72.2 ± 3.11 vs. T2 = 72.3 ± 3.12 kg, p = 1.000; T0 vs. T2, p < 0.001). Sex differences in body mass variations across time are reported in Table 3.

**Table 3:** Body mass variations across time points

	Time points	Males	Females	p-value and R^2^m
BW	T0	81.2 ± 12.2	59.2 ± 3.8	P <0.001 R^2^m = 0.002
	T1	79.4 ± 11.9	57.8 ± 3.8	
	T2	80.4 ± 12.7	58.1 ± 3.9	

p-value and marginal R-squared (R^2^m) refer to the main effect of time in the linear mixed model

*T0* Kathmandu pre, *T1* peak altitude, *T2* Kathmandu post

Energy intake was markedly insufficient relative to the predicted TDEE: females (f) (TDEE (kcal/day) = 3158 ± 203, EI (kcal/day) = 1773 ± 312) and males (m) (TDEE (kcal/day) = 4336 ± 649 kcal/day, EI (kcal/day) = 2102 ± 271 kcal/day) consumed, on average, 44% and 52% less energy than their respective estimated requirements, as reported in Table 4.

**Table 4:** Average daily energy and nutrient intake during the trek compared to estimated requirements and reference limits

Energy balance and macronutrients	Males	Females	Limit
TDEE (kcal/day)	4336 ± 649	3158 ± 203	-
EI (kcal/day)	2102 ± 271	1773 ± 312	-
Deficit (%)	52%	44%	-
Lipids (%EI)	41.42 ± 1.87	43.47 ± 2.59	< 35%^1^
SFA (%EI)	12.75 ± 1.11	14.00 ± 1.42	< 10%^1^
MUFA (%EI)	19.06 ± 0.22	21 ± 1.93	
PUFA (%EI)	6.45 ± 0.94	5.78 ± 0.22	5–10%^1^
C (mg/day)	344.5 ± 91.3	305.1 ± 106.7	< 300 mg^1^

*TDEE* Total daily energy expenditure, *EI* Energy Intake, *SFA* Saturated fatty acids, *MUFA* Monounsaturated fatty acids, *PUFA* Polyunsaturated fatty acids, *C* Cholesterol, *%EI* Percentage of total energy intake

^1^(Herrmann et al. 2024)

In contrast, lipid intake exceeded the reference intake (m: 41.42 ± 1.87, f: 43.47 ± 2.59%EI), resulting in an unbalanced macronutrient ratio. At the same time, saturated fatty acids (SFA) (m: 12.75% ± 1.11, f: 14 ± 1.42%EI) and cholesterol (m: 344.5 ± 91.31, f: 305.1 ± 106.66 mg/day) were above the reference limit, whereas polyunsaturated fatty acids (PUFA) (m: 6.45 ± 0.94, f: 5.78 ± 0.22%EI) were adequate. Reference limits are reported in Table 4 (Viscor et al. 2023).

## Discussion

To our knowledge, this is the first study that investigated cardiovascular, metabolic, renal, and hepatic variations during a high-altitude expedition in a longitudinal setting (before, during, immediately after the trek, and after several months). The main findings indicate that high-altitude exercise imposes a selective physiological strain, triggering coordinated adaptations that involve profound hemodynamic regulation, metabolic reorganization, and distinct organ-specific resilience. In particular, some physiological responses are rapidly affected by hypoxia, whereas others are influenced by the duration and degree of exposure, environmental conditions, physical exertion, and preventive treatment.

Hypobaric hypoxia exposure, especially when combined with exercise, induces greater increases in blood pressure and heart rate, reflecting sustained sympathetic activation to manage the elevated O₂ demand. While heart rate responses tend to normalise within a week in response to down-regulation of the heart’s β-adrenergic receptors (Wolfel et al. 1991) and stabilization of the cardiac output (Reeves et al. 1987), the persistence of elevated arterial pressure, driven by an increase in systemic vascular resistance and carotid body activation, supports a role for the autonomic nervous system during prolonged altitude exposure (Hansen and Sander 2003; Calbet 2003; Kumar et al. 2015). Our data indicate immediate adjustments to altitude changes in heart rate (HR) and systolic blood pressure (SAP), whereas diastolic blood pressure (DAP) showed slower regulation during the initial phase of descent. This kinetic decoupling suggests a prolonged systemic vascular resistance elevation, attributable to sustained sympathetic nervous system activation (Parati et al. 2014).

The kidneys are highly vulnerable to hypoxia due to their high metabolic demand and particular anatomical structure (Fu et al. 2016; Evans et al. 2020); at the same time, persistent activation of sympathetic pathways also contributes to renal stress (Arnaud et al. 2023). The estimated glomerular filtration rate (GFR) is typically used to assess renal function reflecting the renal adaptations to chronic hypoxia. Although acute hypoxic exposure tends to reduce GFR (Bestle et al. 2002; Pichler et al. 2008; Haditsch et al. 2015), after the first week of exposure, GFR stabilizes (Bestle et al. 2002; Haditsch et al. 2015) through increasing filtration fraction (Hurtado et al. 2012) in order to counteract the increased viscosity. Moreover, physical exercise triggers a higher sympathetic tone and dehydration, inducing a lower GFR and resulting in a partial recovery of GFR compared to passive exposure. As a consequence, inadequate renal adaptations can result in the onset of high-altitude renal syndrome (Wang et al. 2022). Furthermore, GFR is regulated through a complex interaction of renal hemodynamics (Weinstein and Anderson 2010), muscular adaptation (Deus et al. 2022), and hormonal pathways (Guldan et al. 2024). The low creatinine levels and elevated GFR in our study present a complex physiological condition. While a maintained GFR might theoretically suggest a hemodynamic response to counteract increased blood viscosity, in our cohort this parameter must be interpreted carefully. The concomitant drop in creatinine points toward metabolic interference, muscle catabolism, and the pharmacological effects of acetazolamide, rather than an active protective renal adaptation. In fact, creatinine can be affected by inflammation and negative nitrogen balance as a consequence of physical exercise combined with hypoxia exposure and negative energy balance (De Rosa et al. 2023). Therefore, the observed improvement requires caution for the possibility of overestimated GFR (Thongprayoon et al. 2016).

The urea-to-creatinine ratio (U/C) increased at high altitude and remained elevated for several days following altitude exposure. This increase was attributable to a rise in urea levels, whereas creatinine concentrations reduced. However, serum urea levels are strongly affected by non-renal determinants, including protein catabolism, dietary protein intake, hepatic urea synthesis, and tubular reabsorption (Gounden et al. 2025). High-altitude trekking-induced diuresis and sweating can lead to hypovolemia (Goldfarb-Rumyantzev and Alper 2014; Palubiski et al. 2020), a condition that disproportionately enhances tubular urea reabsorption relative to creatinine (Sklar et al. 1996), resulting in a biochemical pattern of prerenal azotemia (Uchino et al. 2012). Furthermore, elevated urea levels likely reflect increased protein catabolism rather than intrinsic renal dysfunction. Consistent with this, high-altitude exposure significantly affects body composition through skeletal muscle catabolism (Hamad and Travis 2006), particularly above 5,000 m (G and L 2017), where negative energy balance and physical exertion prevail. Our findings, characterized by a drop in body weight and energy intake, support this 'urea response' hypothesis. For the aforementioned reason, while U/C is frequently used to assess kidney injury, GFR estimated through Cystatin C appears to be a more reliable indicator of renal function under hypoxic conditions (Pichler et al. 2008).

Hypobaric hypoxic exposure also caused alterations in the plasma protein fraction. The A/G ratio decreased at peak altitude and remained reduced early post-hypoxic exposure, driven by decreases in ALB and increases in GLOB levels, respectively. Albumin is sensitive to energy availability, and its pattern may reflect a reorganization of metabolic priorities when intense physical activity and negative energy balance are combined (Wada et al. 2017), rather than alterations in hepatic function. Conversely, the transient GLOB increase may indicate an immune activation in response to hypoxic and physical stress (Morabito et al. 2016).

Within this metabolic framework, dietary intake resulted in a reorganization of the nutrient balance. Despite the hypocaloric setting, participants assumed a relatively high amount of total energy from lipids, accounting for approximately 40% of total energy intake. Consequently, SFA and cholesterol intakes exceeded recommended limits, whereas PUFA intake remained adequate. Despite this, total cholesterol and both LDL and HDL cholesterol concentrations decreased during high-altitude exposure. While the reductions in total cholesterol and LDL are widely reported in the literature (Férézou et al. 1988; Verratti et al. 2015; Karpęcka-Gałka and Frączek 2024), the underlying mechanisms remain debated, and the HDL response is even less clear. For instance, Férézou et al., who first investigated metabolic responses during a high-altitude expedition, found no correlation between the LDL drop and either exercise or body weight changes. Conversely, Karpęcka-Gałka et al. attributed the reduction in total cholesterol and LDL to intense physical exercise combined with a favorable diet, specifically characterized by adequate fiber and elevated MUFA and PUFA intake compared to SFA, alongside a recorded decrease in HDL. However, unlike Karpęcka-Gałka et al., both Férézou and Verratti reported no changes in HDL values. In our study, the concurrent reduction in total cholesterol, LDL, and HDL occurred despite a diet high in SFA. This finding suggests that lipid metabolism under prolonged hypoxia is not primarily driven by fiber intake or dietary fat quality, but rather by a specific metabolic response induced by exercise in hypoxic conditions.

Moreover, the stability of CK, AST, and ALT levels throughout the study suggests that the muscular and hepatic systems were not significantly compromised by trek with similar high-altitude profile. The different systemic responses to hypobaric hypoxia offer direct practical applications; the observed metabolic stress, the renal and hemodynamic changes emphasize the importance of proper dietary choices, adequate hydration and blood pressure monitoring. On the other hand, the preserved hepatic and muscular stability confirms the safety of a well-acclimatized ascent. Finally, our work demonstrated the PoC's suitability in remote high-altitude settings as a practical alternative to classic laboratory methods. For these reasons, these data may guide future studies aimed at developing targeted protective interventions, possibly through the kinetic behavior of the specific markers identified in our work.

### Limitations

This study has several limitations. First of all, despite Acetazolamide prophylaxis standardizing the cohort, it potentially confounded the intrinsic homeostatic response to hypoxia. Consequently, observed renal adaptations, particularly GFR and U/C variations, reflect a combination of physiological acclimatization and pharmacodynamic effects. Moreover, the use of creatinine to estimate GFR may be influenced by the observed reduction in body mass and potential skeletal muscle catabolism (De Rosa et al. 2023). Finally, for logistical reasons, the follow-up phase was completed for 9 of 21 participants.

A potential limitation of this study is the lack of specific validation for the PoC device in extremely high-altitude field settings. Despite the extensive validation of both capillary blood analysis and the specific PoC technology in standard settings, there is a theoretical possibility that high-altitude features, such as cold temperatures and hypobaric hypoxia, could introduce potential bias. However, rigorous procedures were implemented to mitigate these risks, including operator training, analysis in heated and controlled settings, and strict adherence to the device's automated self-calibration and error-detection protocols.

## Conclusion

This is the first study to provide a comprehensive overview of the physiological responses occurring during both the ascent and descent phases of a high-altitude expedition and after a follow-up period.

Overall, these findings indicate that subacute high-altitude trekking is associated with selective, largely reversible multisystemic adjustments rather than uniform systemic injury. Nevertheless, the renal-related changes should be interpreted with caution, as they may reflect the combined influence of hypoxia, exercise, acetazolamide use, and body mass loss rather than a direct protective renal hemodynamic adaptation. The remarkable stability of CK, AST, and ALT proves that, with a gradual ascent, physiological stress is not uniformly distributed but rather highly organ-specific. Finally, the full recovery of all markers during follow-up confirms these changes are adaptive and non-pathological.

In conclusion, high-altitude exposure induces interconnected, organ-specific shifts. A multidisciplinary approach is essential to capture this complex dialogue between systems, moving beyond isolated markers to understand true human acclimatization.

## Data Availability

The datasets collected during and/or analysed during the current study are available from the corresponding author on request.
